# The effects of self-video feedback on the eyedrop instillation techniques of glaucoma patients: a prospective randomized controlled trial

**DOI:** 10.1007/s10792-024-02941-0

**Published:** 2024-02-05

**Authors:** Tae-Eun Lee, Youngri Cho, Hyo Hyun Yoo

**Affiliations:** 1 Department of Ophthalmology, Jeonbuk National University Medical School, 20, Geonjiro Deokjin gu, Jeonju-si, Jeollabuk do 54907 Korea; 2 Department of Medical Education, Jeonbuk National University Medical School, 20, Geonjiro Deokjin gu, Jeonju-si, Jeollabuk do 54907 Korea

**Keywords:** Adherence, Education, Glaucoma, Instillation, Self-video feedback

## Abstract

**Background:**

To evaluate the effectiveness of instillation technique education using self-video feedback in glaucoma patients.

**Methods:**

Sixty-two patients who self-instilled glaucoma eyedrops were randomly assigned to the self-video feedback and control groups according to the block randomization. Each group of the patient was asked to instill eyedrops, and videos were recorded. For the control group, only an educational video was provided. In the self-video feedback group, the patients provided educational video and feedback using a recorded video of their own instillation. After 1 month of education, the patient's instillation techniques were video-recorded again. We divided the steps of instilling eyedrops into ten steps and evaluated whether each step was properly performed using the recorded images from each patient. The main outcome was the proportion of patients who properly instilled their eyedrops in each step.

**Results:**

Before education, there was no significant difference in the proportion of patients who were properly instilled between the two groups. In the group that received video feedback, the proportion of patients who instilled the eyedrops correctly after education in some items was significantly higher than that of the control group, and in particular, the educational effect of 'avoids touching dropper to eyelid or eyelash' was superior.

**Conclusions:**

In patients with glaucoma, education on the method of instillation was effective in improving the techniques of instillation. In the items that required accurate actions, the video feedback that allowed the patient to observe themselves had a better improvement effect compared to the traditional education method.

**Trial registration number:**

KCT0008090 (09/01/2023, retrospectively registered).

## Background

Medication adherence in patients with glaucoma is important. Clinicians rely on patients to self-instill eyedrops and evaluate the effectiveness of medications by measuring the intraocular pressure (IOP) and other parameters. Problems with adherence increase not only the risk of disease progression [1, 2], but also unnecessary drug use, the risk of side effects, and socioeconomic burden. Non-adherence is more problematic in patients with chronic diseases such as glaucoma [3, 4].

Non-adherence may be intentional or unintentional [5]. Intentional non-adherence occurs when patients refuse to take medication or do not fill prescriptions because they feel well or think that they do not need medication. Unintentional non-adherence is caused by external factors (economic burden, physical limitations, or forgetfulness) and incorrect drug administration. Inappropriate eyedrop instillation is unintentional non-adherence but can improve with education, as explored in several previous studies [6–10]. Patients were either directly instructed by clinicians or given handouts or videos. Such education was effective in some studies but not in others. Therefore, a new method is required for this purpose.

Thus, we educated glaucoma patients by recording eyedrop self-instillation and giving feedback; the clinician and patient watched the video together. After 1 month, we explored whether instillation improved more in patients who received video feedback than in those who did not.

## Methods

This study was approved by the Institutional Review Board of Jeonbuk National University Hospital (IRB no. 2020-09-021-004). The procedures conformed to all relevant tenets of the Declaration of Helsinki and all patients provided written informed consent. We included patients with glaucoma or ocular hypertension who had been using glaucoma eyedrops for at least 1 month. The excluded patients were those with a best-corrected visual acuity (VA) poorer than 20/400 in the better eye, those who did not self-instill eyedrops, those who required glaucoma surgery, and those with a systemic disease (such as arthritis or tremors) that might compromise self-instillation.

We asked patients to bring their eyedrops to the clinic and to instill them as usual in a dedicated room with a sink for hand washing, a mirror, clean tissues, and a reclining chair; they were allowed to stand, lie, or sit (depending on their usual instillation method). The entire instillation was video-recorded without intervention by a researcher; the recording was completed when the patient stated. All patients watched videos of appropriate instillation. Block randomization (block size 4; randomization ratio 1:1) was used to assign all participants to one of two groups. Patients in the study group were given additional feedback; the patient and clinician watched the patient-recorded videos together. Such feedback was not provided to the control group. One month later, all patients again self-recorded their instillation at our clinic.

When evaluating the instillation method, we checked whether the 10 steps (Table 1) were correctly performed. The 10 steps of eyedrop instillation used in this study are from previous studies [11, 12] and the information posted on the website of the American Academy of Ophthalmology (AAO) on how to instill eyedrops (https://www.aao.org/eye-health/Treatments/how-to-put-in-eye-drops) were set as items the researchers judged necessary for this study. Among the 10 steps, ‘shake medication gently' evaluated whether the suspension eyedrops user shake the eyedrops before instillation. In the case of solutions eyedrops, ‘shaking’ was not evaluated, but if air bubbles were created due to excessive shaking, it was evaluated as improper use. ‘Squeeze one drop into the inferior fornix’ was evaluated whether too small a dose, or excessive eyedrops exceeding a sufficient dose were used. ‘Wipe away excess eyedrops with tissue ‘ was evaluated as inappropriate use in the following cases: (1) leave the excess eyedrops on face without wiping off, (2) wipe with hands, (3) when the eyedrops are absorbed with the tissue by pressing hard on the eye with a tissue. Evaluations were conducted before and 1 month after education by two researchers (who did not know which group the patients belonged to); if any disagreement arose, it was resolved by discussion.

**Table 1:** The evaluation items

Evaluation items
1. Wash hands
2. Shake medication gently (if needed)
3. Open bottle cap cleanly
4. Tilt head backward and look up when administering eyedrops
5. Pull down lower eyelid to form a pocket
6. Avoid contact between the dropper and the eyelid or eyelash
7. Squeeze one drop into the inferior fornix
8. Gently close eyes for at least 2 min
9. Apply pressure to the punctum
10. Wipe away excess eyedrops with tissue

Patient age, sex, uncorrected distance VA, corrected distance best VA, and uncorrected near VA were collected at the time of enrollment. IOP was measured by a researcher using a Goldmann applanation tonometer (Haag-Streit, Koniz, Switzerland). As the eyedrops lowered IOP, all measurements were taken prior to video-recorded instillation.

We compared the proportions of patients (who received video feedback and not) who performed each of the 10 steps correctly before and after education. Assuming that the proportion who would instill appropriately after education would increase by 20%, a sample size calculation revealed that 31 patients/group would reveal this with an alpha error of 0.05 and a power of 0.80. Randomization was checked using the chi-squared test to compare categorical variables, and the independent Student’s *t* test was used to compare continuous variables. The McNemar test was used to compare patients before and after education (in either group), and the chi-square test was used to compare the two groups. The significance level was set at *P* < 0.05. All statistical analyses were performed using the IBM SPSS Statistics version 18 (IBM Corp., Armonk, NY, USA).

## Results

**Fig. 1:**
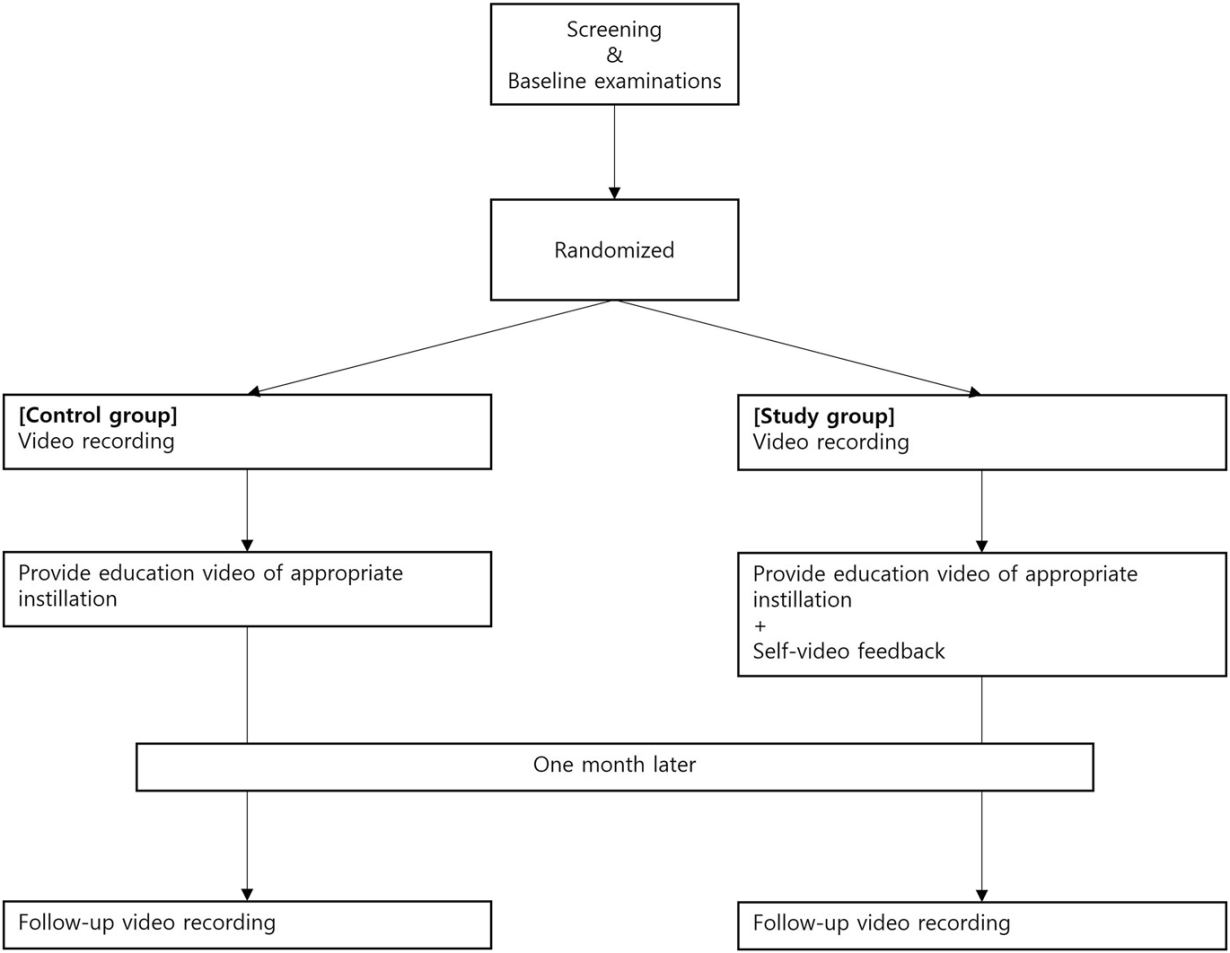
Flow diagram of this study

Eligible participants were recruited as illustrated in Fig. 1. Patient allocation was well balanced. Sex, age, IOP, distance or uncorrected near VA, corrected distance VA, and mean deviation did not differ between the two groups (Table 2). The mean age of the control group was 62.0 ± 14.6 years and that of the study group was 58.8 ± 16.4 years. Males constituted 45.5% of the control group and 51.5% of the study group.

**Table 2:** Demographics

Variables	Control group (*N* = 31)	Study group (*N* = 31)	*P* value
Sex	14:17	16:15	0.800
Age (years)	62.0 ± 14.6	58.8 ± 16.4	0.412
IOP (mmHg)			
Better eye	13.5 ± 2.0	14.3 ± 2.1	0.129
Worse eye	13.0 ± 1.9	14.1 ± 2.5	0.100
Uncorrected distance VA (logMAR)			
Better eye	0.27 ± 0.39	0.26 ± 0.44	0.925
Worse eye	0.46 ± 0.46	0.40 ± 0.54	0.622
Corrected distance VA (logMAR)			
Better eye	− 0.00 ± 0.11	− 0.02 ± 0.09	0.373
Worse eye	0.11 ± 0.22	0.09 ± 0.24	0.708
Uncorrected near VA (logMAR)			
Better eye	0.48 ± 0.40	0.44 ± 0.34	0.684
Worse eye	0.64 ± 0.42	0.59 ± 0.45	0.686
Mean deviation (dB)			
Better eye	− 5.50 ± 5.10	− 4.08 ± 4.81	0.249
Worse eye	− 9.45 ± 5.86	− 8.65 ± 8.76	0.664

Table 3 summarizes the evaluations before education. In terms of cleanly opening the bottle cap (item 3), only one patient held the tip of the bottle by hand after opening the cap; thus, a statistical analysis could not be performed. There was no significant difference between the control and study groups for any of the other evaluation items. In both groups, fewer than 50% of patients performed the other steps correctly with the exceptions of ‘open bottle cap cleanly' and ‘tilt head backward and look up when administering eyedrops.’

**Table 3:** The proportions of patients who appropriately instilled eyedrops before education

Evaluation items	Control group (%) (*N* = 31)	Study group (%) (*N* = 31)	*P* value*
1. Wash hands	16.2	9.7	0.451
2. Shake medication gently (if needed)	6.5	16.2	0.230
3. Open bottle cap cleanly	96.8	100.0	N/A
4. Tilt head backward and look up when administering eyedrops	93.5	90.3	0.451
5. Pull down lower eyelid to form a pocket	41.9	51.6	0.457
6. Avoid contact between the dropper and the eyelid or eyelash	32.3	22.6	0.398
7. Squeeze one drop into the inferior fornix	35.4	19.7	0.211
8. Gently close eyes for at least 2 min	29.1	22.6	0.566
9. Apply pressure to the punctum	6.5	9.7	0.642
10. Wipe away excess eyedrops with tissue	16.2	12.9	0.720

*Chi-squared test

After 1 month of education, the proportion of patients who appropriately instilled eyedrops increased in both groups (Table 4). In the control group, significant differences before and after education were evident for all steps except 'open bottle cap cleanly,’ 'tilt head backward and look up when administering eyedrops’, and 'avoid contact between the dropper and the eyelid or eyelash. In the study group (unlike in the control group), the score for the latter item improved.

**Table 4:** Changes in the proportions of patients who appropriately instilled eyedrops before and after education

Evaluation items	Control group (*N* = 31)			Study group (*N* = 31)		
	Before (%)	After (%)	*P* value*	Before (%)	After (%)	*P* value*
1. Wash hands	16.2	64.5	< 0.001	9.7	74.2	< 0.001
2. Shake medication gently (if needed)	6.5	38.7	0.006	16.2	71.0	< 0.001
3. Open bottle cap cleanly	96.8	100.0	N/A	100.0	100.0	N/A
4. Tilt head backward and look up when administering eyedrops	93.5	93.5	1.000	90.3	93.5	0.375
5. Pull down lower eyelid to form a pocket	41.9	71.0	0.013	51.6	93.5	< 0.001
6. Avoid contact between the dropper and the eyelid or eyelash	32.3	41.9	0.289	22.6	71.0	0.001
7. Squeeze one drop into the inferior fornix	35.4	54.8	0.039	19.7	83.9	0.013
8. Gently close eyes for at least 2 min	29.1	64.5	0.004	22.6	61.3	< 0.001
9. Apply pressure to the punctum	6.5	35.5	0.004	9.7	48.4	0.002
10. Wipe away excess eyedrops with tissue	16.2	41.9	0.012	12.9	74.2	< 0.001

*McNemar test

Table 5 summarizes the between-group differences after education. For the following five steps, the proportions of patients in the study group who performed correctly were significantly higher than in the control group: ‘Shake medication gently,’ ‘pull down lower eyelid to form a pocket,’ ‘avoid contact between the dropper and the eyelid or eyelash,’ ‘squeeze one drop into the inferior fornix,’ and ‘wipe away excess eyedrops with tissue.’

**Table 5:** The proportion of patients who correctly instilled eyedrops after education

Evaluation items	Control group (%) (*N* = 31)	Study group (%) (*N* = 31)	*P* value*
1. Wash hands	64.5	74.2	0.602
2. Shake medication gently (if needed)	38.7	71.0	0.014
3. Open bottle cap cleanly	100.0	100.0	N/A
4. Tilt head backward and look up when administering eyedrops	93.5	93.5	1.000
5. Pull down lower eyelid to form a pocket	71.0	93.5	0.011
6. Avoid contact between the dropper and the eyelid or eyelash	41.9	71.0	0.048
7. Squeeze one drop into the inferior fornix	54.8	83.9	0.037
8. Gently close eyes for at least 2 min	64.5	61.3	0.800
9. Apply pressure to the punctum	35.5	48.4	0.314
10. Wipe away excess eyedrops with tissue	41.9	74.2	0.013

*Chi-squared test

## Discussion

We found that education improved eyedrop intake. Feedback on a self-recorded video of instillation was more effective than simply watching a video on how to instill eyedrops. This was particularly the case when a step required precision, such as ‘avoid contact between the dropper and the eyelid or eyelash.’

Incorrect eyedrop application increases medication waste, costs, traumatic ocular surface damage, and side effects, thus reducing treatment effectiveness and patient satisfaction. According to the expectations of clinicians, up to 80% of patients incorrectly self-administer eyedrops [13]. Although clinicians are aware of the need for education, this is difficult to achieve. Carpenter et al. [10] found that only 34% of glaucoma patients were educated (in clinics) on how to instill eyedrops, and most received only verbal instructions. Several pharmaceutical companies provide instructions or brochures explaining how to properly administer eyedrops, but patients, especially older patients, may not be receptive to this form of information. The cited authors found no correlation between education and improved instillation and hypothesized that this was because patients rarely offered a demonstration of the correct technique. Therefore, a new educational method is required.

It is known that patient self-reports are over-optimistic [9, 14–16]; patients consider that their instillation techniques are better than in fact the case. In a previous study, Davis et al. reported a preference for video education on eyedrop instillation in glaucoma patients [17]. However, it is difficult to improve instillation using handouts or videos if patients do not accept that their techniques are incorrect. The self-video feedback used here overcame this limitation; each patient was shown what was wrong with her technique.

Self-video feedback is often used in skill training, which improves not only skills but also self-confidence [18, 19]. Simple actions such as ‘wash hands’ can be conventionally taught, but video feedback is better when a step requires precise movements, such as ‘avoid contact between the dropper and the eyelid or eyelash' or ‘squeeze one drop into the inferior fornix.’ When instilling eyedrops, accurately instilling an appropriate dose into the eye and preventing contamination of the tip of the bottle is an important step to prevent side effects. However, previous studies have shown that only 28% of patients perform all of these steps correctly [14]. The self-video feedback method is expected to be helpful in instillation education by improving the errors made by patients at these steps better than existing methods.

Our study has certain limitations. First, we did not consider patient education level, socioeconomic status, or severity of glaucoma; these factors affect the level of disease understanding and proper drug use [16]. Additionally, the clinical environment differed from that of the home. In addition, video recording may create nervousness, and a patient may make mistakes when trying to perform better than usual. Finally, the time interval between the (two) evaluations was short. Further studies on the long-term effects of education are required. However, despite these limitations, our new method of self-video feedback was better than existing methods in terms of improvements in certain steps. Evaluations using video recordings have several strengths. This is more objective than existing methods that use questionnaires or otherwise rely on patient responses, and it is possible to repeatedly play, stop, and zoom the video.

We used self-video feedback to improve the instillation techniques in patients with glaucoma. Traditional education has improved some (but not all) steps in the technique. Self-video feedback is more effective than traditional education. Poor patient instillation techniques can lead to drug wastage, dropper contamination, and ocular surface damage. We believe that self-video feedback appropriately evaluates the instillation method and improves instillation techniques.

## Data Availability

The datasets used and/or analyzed during the current study available from the corresponding author on reasonable request.
